# Fine Structure of Tibetan Kefir Grains and Their Yeast Distribution, Diversity, and Shift

**DOI:** 10.1371/journal.pone.0101387

**Published:** 2014-06-30

**Authors:** Man Lu, Xingxing Wang, Guowei Sun, Bing Qin, Jinzhou Xiao, Shuling Yan, Yingjie Pan, Yongjie Wang

**Affiliations:** 1 Laboratory of Quality and Safety Risk Assessment for Aquatic Products on Storage & Preservation (Shanghai), Ministry of Agriculture, Shanghai, China; 2 College of Food Science and Technology, Shanghai Ocean University, Shanghai, China; 3 Institute of Biochemistry and Molecular Cell Biology, University of Goettingen, Goettingen, Germany; Institut de Genetique et Microbiologie, France

## Abstract

Tibetan kefir grains (TKGs), a kind of natural starter for fermented milk in Tibet, China, host various microorganisms of lactic acid bacteria, yeasts, and occasionally acetic acid bacteria in a polysaccharide/protein matrix. In the present study, the fine structure of TKGs was studied to shed light on this unusual symbiosis with stereomicroscopy and thin sections. The results reveal that TKGs consist of numerous small grain units, which are characterized by a hollow globular structure with a diameter between 2.0 and 9.0 mm and a wall thickness of approximately 200 µm. A polyhedron-like net structure, formed mainly by the bacteria, was observed in the wall of the grain units, which has not been reported previously to our knowledge. Towards the inside of the grain unit, the polyhedron-like net structures became gradually larger in diameter and fewer in number. Such fine structures may play a crucial role in the stability of the grains. Subsequently, the distribution, diversity, and shift of yeasts in TKGs were investigated based on thin section, scanning electron microscopy, cloning and sequencing of D1/D2 of the 26S rRNA gene, real-time quantitative PCR, and *in situ* hybridization with specific fluorescence-labeled oligonucleotide probes. These show that (i) yeasts appear to localize on the outer surface of the grains and grow normally together to form colonies embedded in the bacterial community; (ii) the diversity of yeasts is relatively low on genus level with three dominant species – *Saccharomyces cerevisiae*, *Kluyveromyces marxianus*, and *Yarrowia lipolytica*; (iii) *S. cerevisiae* is the stable predominant yeast species, while the composition of *Kluyveromyces* and *Yarrowia* are subject to change over time. Our results indicate that TKGs are relatively stable in structure, and culture conditions to some extent shape the microbial community and interaction in kefir grains. These findings pave the way for further study of the specific symbiotic associations between *S. cerevisiae* and *Lactobacillus* bacteria in TKGs.

## Introduction

Tibetan kefir grains (TKGs), the so-called Tibetan mushroom, are unique dairy starters in Tibet, China, and belong to the same kefir family originating from the Caucasian mountains [1]. TKGs commonly contain lactic acid bacteria (primarily *Lactobacillus* and *Lactococcus*), yeasts, and occasionally acetic acid bacteria within a protein-lipid-polysaccharide solid matrix [2]. Tibetan kefir (TK) is a fermented milk made using TKGs. It is also a kind of popular yogurt in Tibet, which is characterized by a considerably acidic and somewhat alcoholic taste, a slightly yeasty flavor, and very creamy consistency [1], [3]. Although it is a traditional beverage in Tibet, currently TK is becoming a kind of home-made yogurt in numerous families across the mainland of China. Similar kefir beverages have been found in other regions and countries, e.g., Taiwan, Russia, Turkey, Brazil, Argentina, Korea, Germany, Ireland, Portugal, and France [4]–[13]. In addition, besides its higher nutritional value, kefir appears to possess dramatic health benefits including enhancing the immune system, improving digestive health, as well as having antimicrobial, antitumoral, and antioxidant effects [14]–[18].

To give insights into the diversity of microflora in TK and TKGs, culture-dependent and culture-independent methods have been taken into consideration [2], [3], [19]. Zhou et al. investigated microbial communities in TKGs by denaturing gradient gel electrophoresis followed by sequencing of the majority of bands [2]. They found that the dominant bacterial species were *Pseudomonas* sp., *Leuconostoc mesenteroides*, *Lactobacillus helveticus*, *Lactobacillus kefiranofaciens*, *Lactobacillus casei*, *Lactococcus lactis*, and *Lactobacillus kefiri*; the dominant yeasts were *Kazachstania unispora*, *Kluyveromyces marxianus*, *Saccharomyces cerevisiae*, and *Kazachstania exigua* [2]. Recently, Gao et al. analyzed the bacterial diversity of TKGs from different areas in China using the metagenomic methods, and observed that the TKGs consisted of *Lactococcus*, *Lactobacillus*, *Acetobacter*, *Shewanella*, *Leuconostoc*, *Pseudomonas*, *Streptococcus*, *Acinetobacter*, *Pelomonas*, *Dysgonomonas*, and *Weissella* [3], [19].

Thus far, the overall community composition of bacteria in TKGs is highlighted [3], [19]. In contrast, the inventory of the yeasts associated with the TKGs is far from complete [2], [3]. Yeasts play a vital role in the symbiosis of TKGs, which makes it essential to investigate the distribution and diversity of yeasts in TKGs. Additionally, besides the description of its cauliflower-like appearance, the fine structure of TKGs has never been studied thoroughly, making it challenging to shed light on the symbiotic association between the yeasts and bacteria in TKGs.

The aim of this study was to investigate (i) the fine structure of TKGs by using a stereomicroscope and epifluorescence microscope combined with the thin section technique, and (ii) the distribution, diversity, and shift of yeasts in TKGs with a scanning electron microscope (SEM), culture-independent 26S rRNA gene cloning and sequencing, real-time quantitative PCR as well as *in situ* hybridization with specific fluorescence-labeled oligonucleotide probes (fluorescence *in situ* hybridization, FISH).

Our results indicate that a single TKG unit has a hollow globular structure with a diameter between 2.0 and 9.0 mm, containing a polyhedral net structure formed mainly by bacteria. Three yeast species – *S*. *cerevisiae*, *K. marxianus*, and *Yarrowia lipolytica* – are dominant in TKGs and are commonly present on the outer surface of TKGs. *S*. *cerevisiae* is the stable dominant yeast species, while the composition of *Kluyveromyces* and *Yarrowia* are subject to change over time.

## Materials and Methods

### Sample and culture of TKGs

TKGs were offered originally by a private household that agreed to use them in this study. In the laboratory, the grains were cultured at 25°C in pasteurized whole milk (Bright Dairy, Shanghai) which was renewed every 2 days. The grains were maintained in the laboratory in two distinct groups of naturally cultured TKGs (NCTKGs) and aseptically cultured TKGs (ACTKGs). The NCTKGs suffered potential contamination from environmental microorganisms. In contrast, the ACTKGs were cultured in sterile beaker covered with 8 layers of sterile gauze and washed using sterile water in clean bench in case of changing the milk. Samples of NCTKG 1 and ACTKG 1 were taken after the household TKGs had been cultured in the laboratory for one month.

### Stereomicroscope observation

TKGs were filtered and washed several times using sterile water to remove clotted milk adhered to the surface of the grains. Then they were kept in Petri dishes and directly observed under the stereomicroscope (Discovery V20, ZEISS, Germany).

### Frozen sections and SYBR Green I staining

Grains (n = 30) were embedded in the tissue freezing medium (SAKURA, USA), which was frozen at −20°C for 10 min in the freezing microtome (CM1950, Leica, Germany). Subsequently, the frozen grains were cut successively into sections of 3 µm thickness in the order of outer to the inner surface. After being fixed in 4% formaldehyde for 14 h and stained with 25× SYBR Green I (Solarbio, Beijing, China) for 15 min, the thin sections were observed under an epifluorescence microscope (Zeiss Axiophot, Germany).

### SEM observation

TKGs (n = 12 for both the NCTKGs and ACTKGs) were suspended in 0.1 M phosphate buffer for 2 min. They were then exposed in 2.5% cold glutaraldehyde solution to rinse for 30 min and subsequently immediately stored in 2.5% glutaraldehyde solution for 16 h at 4°C. Afterwards, the samples were washed in 0.1 M phosphate buffer three times, for 15 min each time, and then postfixed in 1% osmium tetroxide for 1 h at 4°C, followed by washing in 0.1 M phosphate and a series of ethanol for 10 min at room temperature.

The fixed TKGs were desiccated in isoamyl acetate twice (for 15 min each time) and dried in a critical-point dryer (HCP-2 HITACHI, Japan) for 5 h. Then, the dehydrated samples were displayed on specimen stubs with double-sided adhesive tape. After coating with gold with a sputter coater (Cressingtom 108 auto), the samples were observed using a SEM (S-4800 HITACHI, Japan).

### Culture-independent cloning and sequencing of 26S rRNA gene

For each sample, three pieces of the grain materials, a total of approximately 40 mg, were aseptically and randomly taken from a single grain unit, pestled with TissueRuptor (TIANGEN, Beijing, China), and then suspended in 600 µl of 1.2 M sorbitol buffer. After the addition of 50 U lyticase, the samples reacted for at least 30 min at 30°C. The samples were centrifuged at 6000×*g* for 10 min, and the precipitate was resuspended in the buffer (20 mM Tris-HCl, 2 mM sodium EDTA, 1.2% Triton X-100) with 20 mg/ml lysozyme for 50 min at 37°C. Then the total DNAs were extracted according to the instructions of the DNeasy Blood and Tissue Kit (Qiagen, Hilden, Germany) with a modification of adding 40 µl Proteinase K solution.

The D1/D2 domain of the 26S rRNA gene of yeast was amplified using the primer pair NL1 and NL4 [20] (Table 1). PCR was performed in a total reaction volume of 50 µl containing 25 µl of 2× *Taq* PCR MasterMix (Tiangen, Beijing, China), 1 µl of 10 µM of each primer, and approximately 100 ng DNA template. The PCR program was as follows: 94°C for 5 min, followed by 35 cycles of 94°C for 30 s, 55°C for 30 s, 72°C for 1 min, and a single final extension step of 72°C for 10 min. The amplified PCR products of D1/D2 domain of 26S rRNA gene fragments were purified using the PCR Fragment Purification Kit (Tiangen, Beijing, China) and then cloned with the pGM-T Cloning Kit (Tiangen, Beijing, China). The recombinant plasmids were transformed into competent *Escherichia coli* T0P 10 cells according to the instructions recommended by the supplier (Tiangen, Beijing, China). Positive clones carrying approximately 600 bp of 26S rRNA gene were identified by colony PCR and 2.0% agarose gel electrophoresis. Positive clones (n = 110) of each sample were sequenced on one strand by using primer T7 (Sangon, Shanghai, China). The obtained sequences were analyzed for sequence similarity by nucleotide BLAST (http://blast.ncbi.nlm.nih.gov/Blast.cgi).

**Table 1:** PCR primers used in this study.

Target group	Primer (5′ to 3′)	Product size (bp)	Reference
**Clone library/Plasmid standard**			
Yeast	NL1: GCATATCAATAAGGGGAGGAAAAG	615	[20]
	NL4: GGTCCGTGTTTCAAGACGG		
**Real-time qPCR**			
Yeast	Yeast-F: GAGTCGAGTTGTTTGGGAATGC	125	[21]
	Yeast-R: TCTCTTTCCAAAGTTCTTTTCATCTTT		
*Saccharomyces*	Sacch-2F: GGTCCGTGTTTCAAGACG	185	This study
	Sacch-2R: TAGGGGAATCTCGCATTTCA		
*Kluyveromyces*	Kluyv-F: TGTTTCAAGACGGGCGGC	188	This study
	Kluyv-R: CAGACATGGCGTTTGCTTCG		
*Yarrowia*	Ylip-F: GAGGGGCGGCTGAAACCTCG	356	This study
	Ylip-R: ACGGCGAGTGAAGCGGCAAA		

### Real-time quantitative PCR

Primers for real-time qPCR are shown in Table 1 [20], [21]. The specific primers were designed based on the cloned 26S rRNA gene sequences using Geneious 5.6.2 software (Biomatters, USA) and Primer 5.0 (Premier, Canada), and their specificity was checked in the GenBank database by using the Primer-BLAST program (http://www.ncbi.nlm.nih.gov/tools/primer-blast/).

Plasmid standards used in this study were prepared as follows. The D1/D2 domain of the 26S rRNA genes were cloned, screened, and sequenced as mentioned above. Afterwards, the positive clones, carrying 26S rRNA gene fragments of *S*. *cerevisiae*, *K*. *marxianus*, or *Y*. *lipolytica*, were cultivated. The recombinant plasmid was extracted by using the QIAprep Spin Miniprep Kit (Qiagen, Hilden, Germany) and quantified with BioTek Synergy 2.0 (BioTek, USA) using Quant-iT PicoGreen dsDNA Reagent and Kits (Invitrogen, USA). The copy number of the 26S rRNA gene of the yeasts was calculated referring to [22].

The total genomic DNA of TKGs was extracted and quantified according to the methods described above. Then a series of dilution was conducted to obtain a final concentration ranging from 0.1 to 1.0 ng/µl. The reaction mix (20 µl) contained 2.0 µl of template (plasmid standard or TKG genomic DNA), 0.4 µM of each specific primer, 10 µl of SYBR *Premix Ex Taq* II (2×) (Takara, China), and 0.4 µl of Rox Reference Dye (50×). The following qPCR thermal program was used: 95°C for 10 min, followed by 40 cycles of 94°C for 15 s, 59°C for 30 s, and 72°C for 30 s. The amplification reaction was performed on an ABI 7500 Fast real-time PCR system along with version 2.0.1 of the software (Applied Biosystems, USA). All of the experiments for quantifying total yeasts, *Kluyveromyces*, *Saccharomyces*, and *Yarrowia* were performed with three parallels and repeated three times. The data were statistically analyzed by using the statistical software package SPSS 17.0 (IBM, USA). The statistical degree of significance was set at a *p* value of <0.01.

### FISH

All probes used in this study are listed in Table 2. The probe Ylip (targeting *Yarrowia*) was referred to [23]. Probes Kluyv 1 and 2, targeting a different region of the 26S rRNA gene of *Kluyveromyces* to enhance the fluorescence signals, and Sacch (targeting *Saccharomyce*s) were designed according to the principles in [24]. Their sequence specificity was checked in the GenBank database by using the Primer-BLAST program (http://blast.ncbi.nlm.nih.gov/Blast.cgi) as well as being based on the purely cultured strains of *K*. *marxianus*, *S*. *cerevisiae*, and the *Y*. *lipolytica* isolated from TKGs.

**Table 2:** FISH probes used in this study.

Probe	Target group	Probe sequence (5′ to 3′)	Fluorescein (5′)	% Formamide	Reference
Kluyv 1	*Kluyveromyces*	CCAACCGCTAAAACTGAT	CY3	20	This study
Kluyv 2		CAGAGCCACATTCCCGAG		20	This study
Sacch	*Saccharomyces*	ACGAGATTCCCCTACCCA	FITC	20	This study
Ylip	*Yarrowia*	CACTCATTTCCTTCCC	CY3	30	[23]

The frozen thin sections were fixed, pretreated, and hybridized as described in [25]. To avoid nonspecific hybridization, a series of hybridization experiments were conducted by varying the concentration of formamide from 5 to 70% in the hybridization buffer and its corresponding concentration in the washing buffer.

### Nucleotide sequence accession numbers

The cloned 26S rRNA gene sequences of yeasts in TKG have been deposited in GenBank under the accession numbers KF809966–KF810103.

## Results

### Fine structure of TKGs

**Figure 1:**
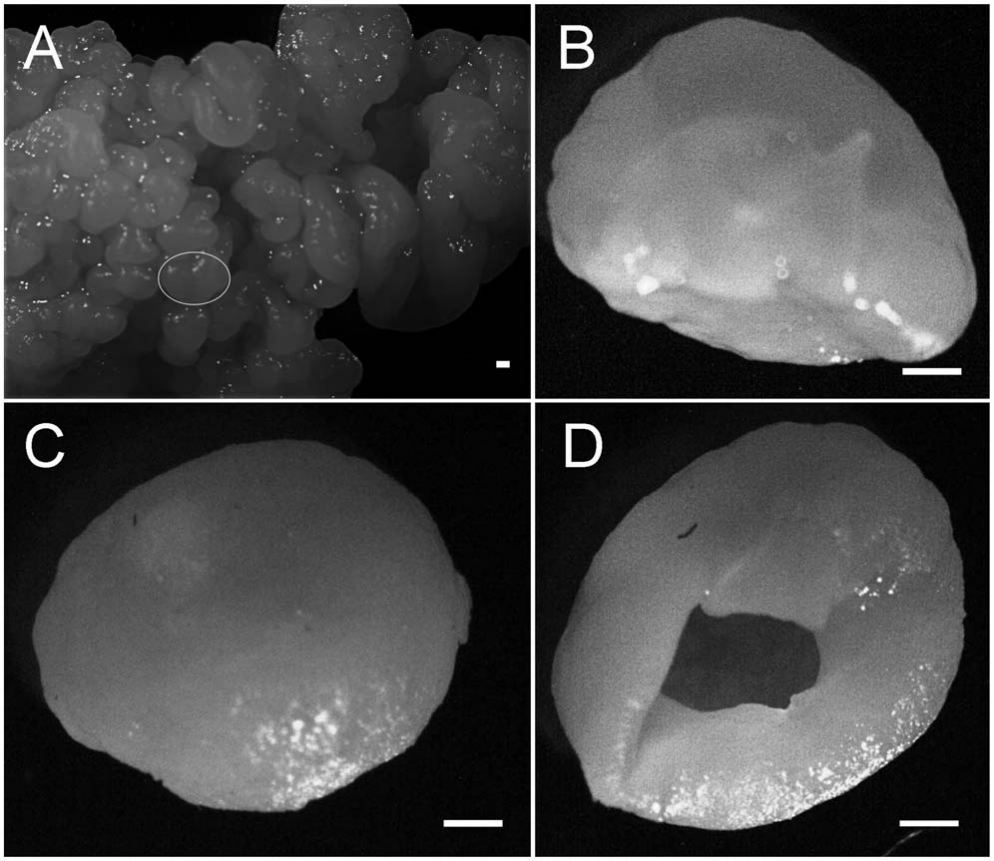
Stereomicroscope images of the Tibetan kefir grains. (A) Tibetan kefir grains and a single grain unit marked with a circle in (A) and observed from the side (B), the top (C), and the bottom (D). Scale bar = 1.2 mm.

**Figure 2:**
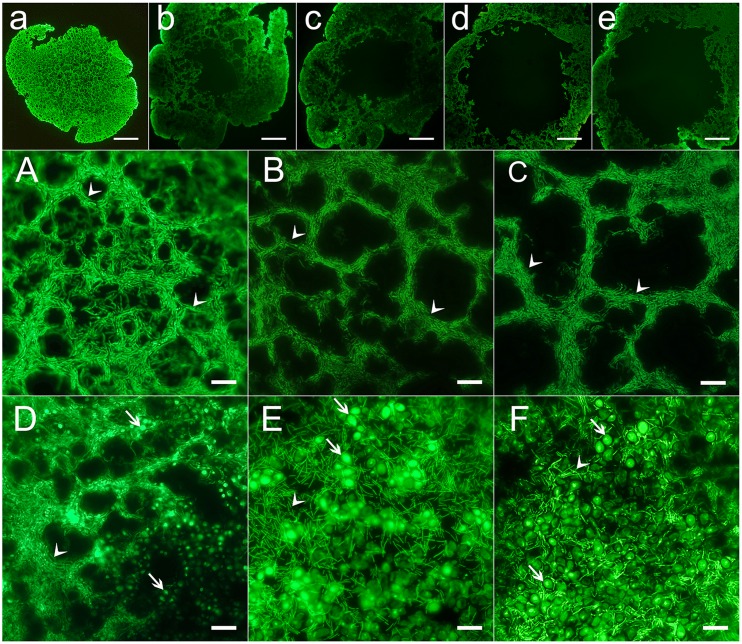
The microflora in TKGs observed on thin sections stained with SYBR Green I. (a–e) Series thin sections, from outside towards inside, of a single hollow globular unit. Scale bar = 500.0 µm. (A–C) The polyhedron-like net structure, from outside towards inside, of a grain unit. (D–F) The distribution of yeasts on the outside wall of a grain unit. The arrows indicate the representative yeast cells. The rod-shaped cells (A–F) are bacteria as marked with the arrowheads. Scale bar = 10.0 µm.

**Figure 3:**
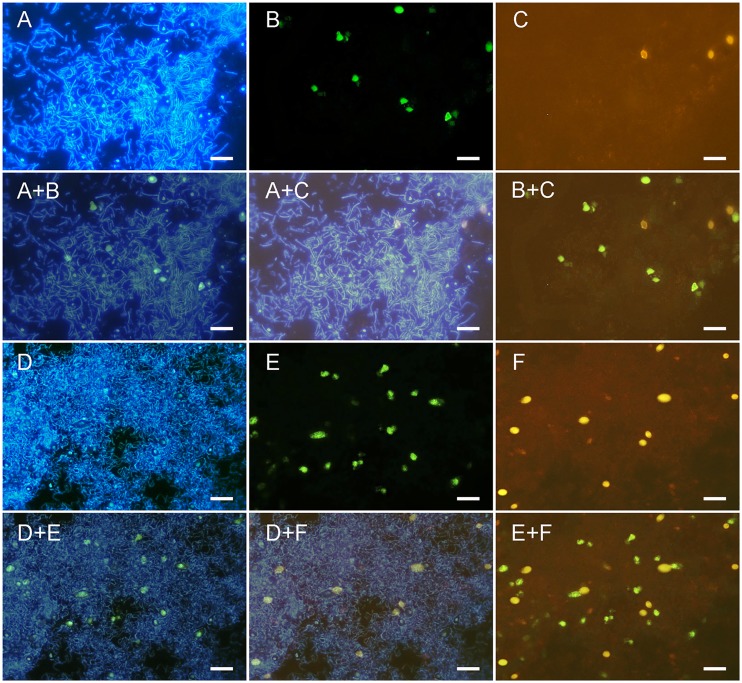
FISH analysis of yeast species in TKGs. Epifluorescence micrographs of bacteria and yeasts stained with DAPI (A, D); *Saccharomyces* hybridized with probe Sacch (B, E); *Kluyveromyces* hybridized with probes Kluyv 1 and 2 (C); and *Yarrowia* hybridized with probe Ylip (F). Others represent merged photographs. The bacilliform signals are the DAPI stained bacteria, and the bright dots are the yeasts. Scale bar = 10.0 µm.

Under the stereomicroscope, the cauliflower-shaped TKGs consisted of numerous small grain units (Figure 1). Each one revealed a hollow globular structure with a diameter commonly between 2.0 and 9.0 mm (Figure 1). The grain unit wall was approximately 200 µm in thickness based on thin section observations. During the cultivation, some of the small grain units were shed and grew into big grains again. On the thin sections of TKGs, the abundant rod-shaped cells hybridized only with the probe Eub338 specific for most Eubacteria (Figure S1 and Figure 2) but not with probes specific for yeasts (Figure 3 and Figure 2), which indicated that they were bacteria; by contrast, the spherical cells hybridized only with the probes specific for yeasts (Figure 3 and Figure 2) but not with the probe Eub338 specific for most Eubacteria (Figure S1), which confirmed that they represented yeasts. Interestingly, a polyhedral-like net structure was observed in the grain unit wall, which was composed almost entirely of bacteria (Figure 2 and Figure S1). Furthermore, along the direction of outer to inner surface, the net structures became gradually larger in diameter and fewer in number (Figure 2). No obvious differences in fine structure were observed between the NCTKGs and ACTKGs.

### Distribution of yeasts in TKGs

To understand the nature of the distribution of yeasts in TKGs, frozen thin sections prepared from the outside to inside surfaces were stained with SYBR Green I and observed under an epifluorescence microscope. As a result, yeasts appeared to normally grow together and to form colonies embedded in the bacterial community (Figure 2). They were mainly present on the outside surface and places close to it (Figure 2). Both yeasts and bacteria showed a decline in density from the outer to the inner surface (Figure 2). In comparison with the number of bacteria, that of yeasts was far less in TKGs, which was in agreement with SEM and FISH observations as well as being confirmed based on qPCR analysis (see below).

**Figure 4:**
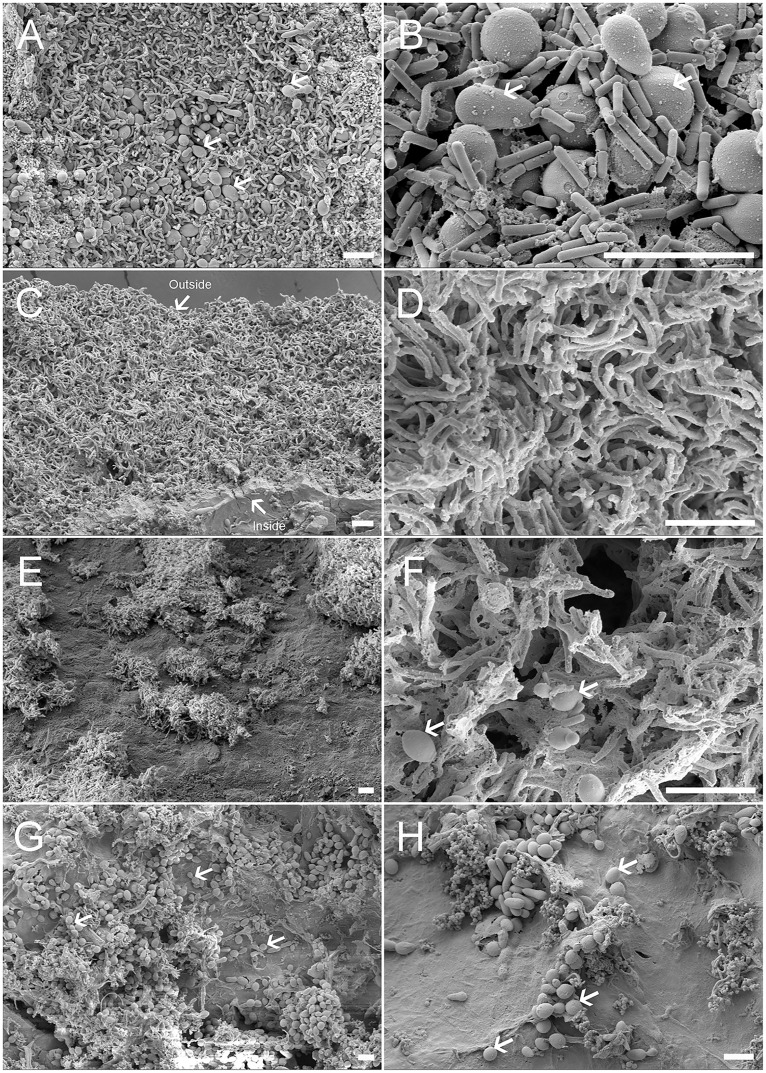
Scanning electron micrographs of TKGs. (A) The outer surface of TKGs; (B) The high magnification of (A); (C) The cross-section of TKGs; (D) The high magnification of (C); (E) The inner surface of NCTKGs; (F) The high magnification of (E); (G) The inner surface of ACTKGs; (H) The high magnification of (G). The arrows, shown in (A), (B), (F), (G), and (H), indicate the representative yeasts in KGs. The outside and inside surfaces of KGs are indicated by the arrows in (C). Scale bar = 10.0 µm.

Based on SEM, the yeasts mainly colonized on the outer surface of TKGs (both the NCTKGs and ACTKGs). The majority of them were spherical (2.0–5.0 µm in diameter) or oval (4.0–8.0×2.0–5.0 µm); occasional club-shaped yeasts (4.0–7.0 µm in length) were also observed (Figure 4). Few yeast cells were observed present in the middle region between the outside and inside surfaces (Figure 4). As for the inner surface, like the observations on the thin sections (Figure 2), both yeasts and bacteria were less dense compared with the outer surface. Interestingly, The ACTKGs clearly had more yeasts on the inner surface than the NCTKGs (Figure 4).

### Identification of yeasts in TKGs based on 26S rRNA gene cloning and sequencing

Four clone libraries of the D1/D2 of the 26S rRNA gene were constructed for NCTKGs (1 and 2) and ACTKGs (1 and 2). NCTKG-2 and ACTKG-2 were the samples cultivated for 10 months more than NCTKG-1 and ACTKG-1. In total, 104, 93, 102, and 106 clones were sequenced successfully for each of the four clone libraries (Table 3). Blast sequence similarity analysis revealed that all cloned sequences shared a similarity of 97 to 100% compared with their correspondingly most closely related sequences in the GenBank database (Table S1). Approximately, 90% of the clones with ≥99% of sequence identity were grouped into three species – *S*. *cerevisiae*, *K*. *marxianus*, and *Y*. *lipolytica* – within three genera – *Kluyveromyces*, *Saccharomyces*, and *Yarrowia*; the remaining 10% with ≥97% of sequence identity belonged to these 3 yeast genera mentioned above as well (Table S1). It suggested that the yeasts in TKGs were comprised mainly of three dominant species and were of relatively low diversity on genus level.

**Table 3:** Phylotypes of yeast community in TKGs.

		Number of clones			
Genus	Species	NCTKG		ACTKG	
		104 (N-1)	93 (N-2)	102 (A-1)	106 (A-2)
*Saccharomyces*	*S*. *cerevisiae*	34	74	30	85
	*Saccharomyces* sp.	10	0	7	1
*Kluyveromyces*	*K*. *marxianus*	45	1	57	4
	*Kluyveromyces* sp.	15	0	8	0
*Yarrowia*	*Y*. *lipolytica*	0	18	0	16

N-1: NCTKG-1, N-2: NCTKG-2, A-1: ACTKG-1, A-2: ACTKG-2. The NCTKG-2 and ACTKG-2 are the samples cultivated for 10 months more than the NCTKG-1 and ACTKG-1. A sequence identify of ≥99% is considered the identification to the species level; that of ≥97% to the genus level [40], [41].

*S*. *cerevisiae* was detected in all four TKG samples and represented the second dominant yeast species in the NCTKG-1 (32.7%, 34/104) and ACTKG-1 (29.4%, 30/102), and was the compulsively dominant one in the NCTKG-2 (79.6%, 74/93) and ACTKG-2 (80.2%, 85/106) (Table 3). *K*. *marxianus* was the most predominant yeast species in the NCTKG-1 (43.3%, 45/104) and ACTKG-1 (55.9%, 57/102). Surprisingly, *Kluyveromyces* was hardly detected in both the NCTKG-2 (1/93) and ACTKG-2 (4/106) 10 months later. Furthermore, *Y*. *lipolytica*, absent in both the NCTKG-1 and ACTKG-1, was found to be present in the NCTKG-2 and ACTKG-2 with relatively high abundance (Table 3) and has not been reported in other kefir grains.

### qPCR analysis of the yeasts in TKGs

**Figure 5:**
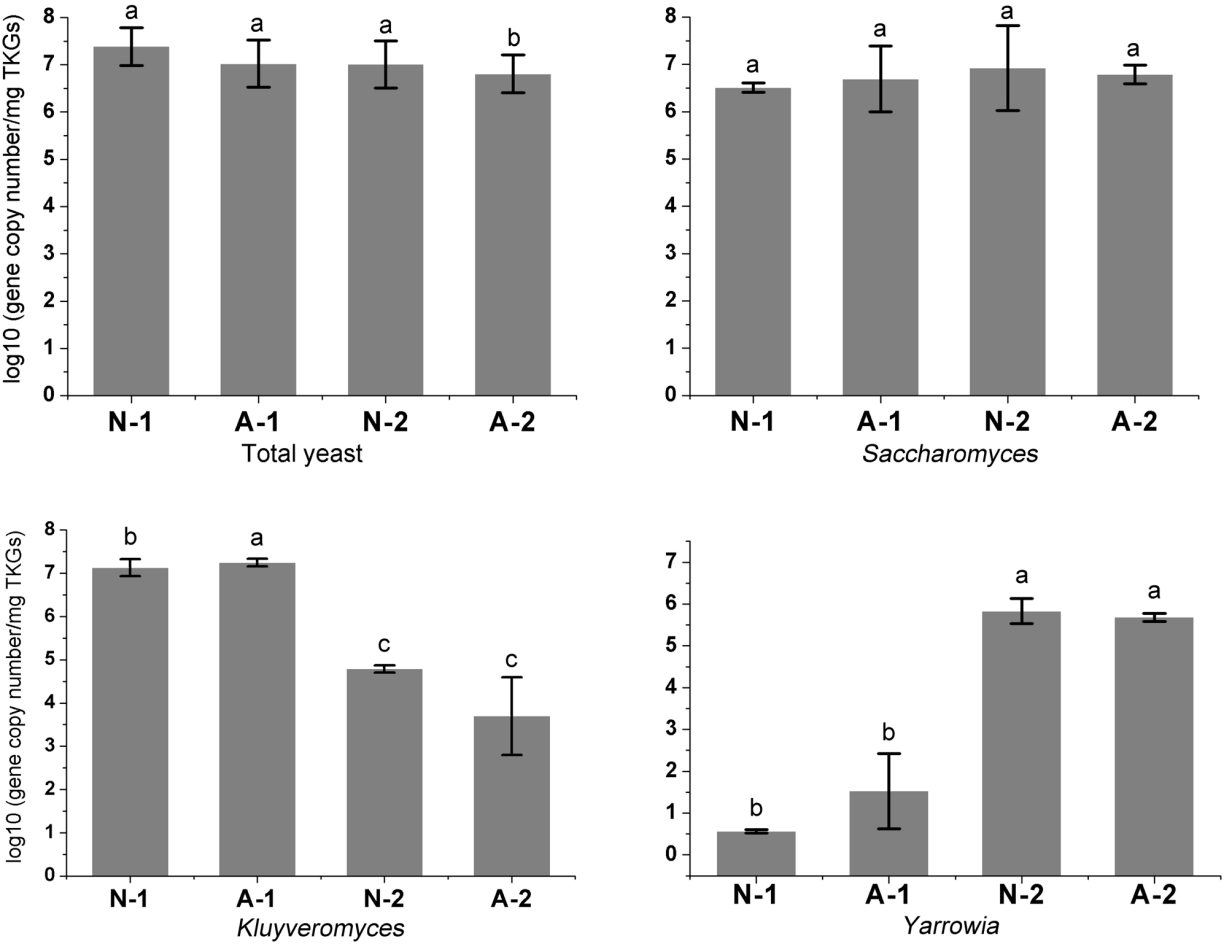
The shift of yeast species in TKGs during a long-term subculture as revealed by real-time qPCR assay. N-1: NCTKG-1, N-2: NCTKG-2, A-1: ACTKG-1, A-2: ACTKG-2. NCTKG-2 and ACTKG-2 are the samples cultivated for 10 months more than NCTKG-1 and ACTKG-1. Standard deviations are shown by error bars. Differing letters within each subfigure (i.e., a, b, c) indicate statistically significant differences (*p*<0.01). The statistical analysis was performed based on the experimental data of 3 repeats, each in 3 parallels, and a total of 9 grains were analyzed for each sample.

As shown in Figure 5, the number of total yeasts demonstrated a slight decrease to 1.02±0.56×10^7^ copy number/mg in the NCTKGs and 6.38±0.49×10^6^ copy number/mg in the ACTKGs. That of *Saccharomyces* did not change significantly during the 10 months of cultivation and only slightly increased to 8.36±0.90×10^6^ copy number/mg in the NCTKGs and 6.10±0.25×10^6^ copy number/mg in the ACTKGs. The most obvious changes were the number of *Kluyveromyces* and *Yarrowia*, which differed significantly (*p*<0.01) during the 10 months of culture (Figure 5). In both the NCTKGs and ACTKGs, the *Kluyveromyces* declined greatly from approximately 1.35∼1.78×10^7^ to 10^3^∼10^4^ copy number/mg. In contrast, the *Yarrowia* increased greatly from nearly zero to the second dominant yeast species with approximately 10^5^ copy number/mg. The shift in the number of *Kluyveromyces* and *Yarrowia* in TKGs, determined by qPCR, was in agreement with the observation in the clone libraries described above.

### FISH analysis of yeasts in TKG

To get a specific hybridization, a series of hybridization experiments were conducted on the thin sections of TKGs by varying the concentration of formamide from 5 to 70% in a hybridization buffer and its corresponding concentration in washing buffer. As a result, the hybridization condition of high stringency of 20% formamide was obtained for probes Kluyv 1 and 2, and Sacch (Table 2), and used in the subsequent experiments. Furthermore, FISH was also performed using the pure culture strains of *S*. *cerevisiae*, *K*. *marxianus*, and *Y*. *lipolytica*. Strong positive signals were only observed in the specific hybridization of probes and their corresponding yeast species (Figure S2), which further confirmed the specificity of the probes and the hybridization conditions.

FISH verified the existence of three genera of yeasts – *Kluyveromyces*, *Saccharomyces*, and *Yarrowia* – in TKGs (Figure 3), which indicated that the DNA extracts originated from TKGs and were free from alien contamination. In general, *Saccharomyces* was the dominant yeast species in TKGs, and these three genera of yeasts appeared to localize together in the same TKG niches instead of being separate in different TKG niches (Figure 3). FISH observations were consistent with those of cloning and qPCR.

## Discussion

### Fine structure of TKGs

TKGs are composed of a polysaccharide/protein matrix, containing a complex microbial community of lactic acid bacteria, yeasts, and sometimes acetic acid bacteria, and represent one of the unusual specific symbiotic systems in nature [1]. Thus far, however, little has been known about the fine structure of the grains, knowledge which is crucial to shed light on the symbiotic relationship between bacteria and yeasts. Our studies confirm that the cauliflower-like shaped TKGs are grown into from a single hollow globular structure unit of TKG and comprise numerous such units as well. Given the fact that TKGs are produced in milk, it is conceivable to speculate that the hollow globular structures contribute to the uptake of nutrients by their designers and residents of bacteria and yeasts as well as to the structural stability of the grains. Coincidently, this idea is further supported by the observation of the polyhedron-like net structures in TKGs, which are formed densely on the outer surface and decrease in density towards the inner surface (Figure 2). Thus far, similar structures have not been observed in other KGs. It has been confirmed that the formation of the polyhedron structure is attributed to the high stability of the S-Au-S structural unit [26]. In addition, the majority of viruses found so far possess icosahedral structure of their capsid shell, which is believed to contribute to its stability [27]. Therefore, the polyhedron-like net structure in TKGs may also play a crucial role in the stability of the grains. Interestingly, this structure seems to result from a long time coevolution of the bacterial and yeast symbionts, since the attempts have failed to reproduce the grains by coculture of the pure culture bacteria and yeast strains isolated from kefir grains [28], [29]. Although the bacteria appear to be the major players responsible for the net structure (Figure 2), the net structure is completely disrupted when the yeasts are removed from the grains (Wang et al., unpublished data). Whether the yeasts are directly involved in forming the polyhedron-like net structure or the structure breaking is due to the death of bacteria resulting from the elimination of their symbiotic partners of yeasts, remains open for further study.

### Diversity of yeasts

Thus far, the bacterial diversity in TKGs has been studied by using culture-dependent and independent methods, e.g., metagenomics and pyrosequencing [2], [3], [19]. Members of *Lactobacillus* turn out to be the most common bacterial species in TKGs, which also holds true to our grains (data not shown) and most other kefir grains [30], [31]. In contrast, as for yeasts in TKGs, their diversity is far from complete [2], [3]. We observed that the cauliflower-like structure of TKGs is friable, and that the bacterial density decease greatly when the yeasts are removed from the grains (Wang et al., unpublished data). This suggests that the bacteria and yeasts share an unknown close symbiotic relationship, and both of them contribute to the formation of the grains. Accordingly, the culture-independent method of cloning and sequencing was initially used to reveal the diversity of the yeast community as well as the predominant yeast species.

Analysis of the 26S rRNA gene clone library showed three genera of yeasts in TKGs – *Saccharomyces*, *Kluyveromyces*, and *Yarrowia*. *Yarrowia* has not been identified in other TKGs and kefir grains (KGs) and may come initially from the house environment where the TKGs used in this study once grew. FISH confirmed that these three yeast lineages originated from the TKGs and not from the environment. Additionally, it also indicates that *Saccharomyces* and *Kluyveromyces* are the dominant yeasts in TKGs. These two kinds of yeasts are also present in TKGs sourced from geographically distinct regions of China [2], [3] and are common yeast species in most other KGs worldwide. Interestingly, although *Kazachstania* is commonly detected in TKGs as well as other KG lineages, it is absent in our samples. On the whole, yeasts were characterized by a high diversity over the whole family of KGs but a relatively low diversity in a single KG [11], [32]–[34]. In addition, like *Yarrowia*, some species are solely associated with one or two lineages of KGs [3], [11]. Taken together, these suggest that certain yeast species in KGs may be subject to change due to the competition of other yeast species present in the grains or to the natural selection by the culture milk with different nutrient ingredients. Since the culture milk used in our study is different from others, it possibly better meets the growth needs of *Yarrowia* than those of *Kluyveromyces*.

### Shift of yeasts

It has been shown that *S. cerevisiae* is an adapted yeast species to grow in a low pH, low dissolved oxygen, and alcohol-rich environment [35], an environment with which the TKs are typically characterized. Recently, Mendes et al. have reported the direct interaction between *S. cerevisiae* and *Lactobacillus delbrueckii* based on transcriptome assay, which indicates that these two partners cooperate well with each other in order to take advantage of carbon and nitrogen resources, and have evolved metabolism strategies to cope with the challenging conditions of low pH and ethanol [36]. In the present study, both the real-time quantitative PCR and clone library assays revealed that the number of total yeasts and *Saccharomyces* yeast remained relatively stable, but *Kluyveromyces* and *Yarrowia* yeasts shifted greatly over the 10 months of subculture of TKGs. This finding suggests that (i) *Kluveromyces* and *Yarrowia* are less resilient to the laboratory culture condition than *Saccharomyces*; (ii) *Yarrowia* is more adapted to the laboratory culture condition than *Kluveromyces*; (iii) *S*. *cerevisiae* may be a more generalist species than *Kluveromyces* sp. and *Yarrowia* sp. as well as play a leading role among other yeast species in the symbiotic associations with the bacteria in the TKGs.

Interestingly, *Kluyveromyces* yeasts decreased heavily but *Yarrowia* yeasts revealed a remarkable increase from nearly zero to being the dominant population. As for *Kluyveromyces*, whose carbon substrate spectrum is much broader than that of *S*. *cerevisiae* [37], they are able to metabolize lactose in milk. Accordingly, *Kluyveromyces* yeasts are the major competitor to *Lactobacillus* bacteria as well as to other yeast species, such as *S*. *cerevisiae* and *Y. lipolytica*, which are unable to utilize lactose directly. However, why did it drop down significantly 10 months later? We speculate that the culture milk was the key cause. The TKGs examined in our study had been grown in skimmed milk by a private householder for years. In our study, given that KGs originated in natural fresh milks, e.g., cow, sheep, goat milk, which contains >3.5% fat, the whole milk instead of skimmed milk was used in the laboratory to cultivate TKGs. Consequently, although it is unable to utilize lactose, *Y. lipolytica* in the grains is able to grow by utilizing fat. Since *S*. *cerevisiae* is able to grow with or without oxygen, but *Y. lipolytica* and *Kluyveromyces* depend on oxygen for growth, *Y. lipolytica* may compete with *Kluyveromyces* but not *S*. *cerevisiae* in the grains. In addition, the fatty acids produced by *Y. lipolytica* may inhibit the growth of *Kluyveromyces* [38]. Taken together, these suggested that *Kluyveromyces* decreased over time due to the competition of *Lactobacillus*, the dominant bacteria in TKGs (data not shown), and *S*. *cerevisiae* and *Y. lipolytica*. It also explained why *Y. lipolytica* was solely observed in this study but not in others using the skimmed milk instead of whole milk for the KG culture.

### NCTKGs and ACTKGs

In comparison with the NCTKGs, the ACTKGs tended to grow faster, and the grains were tighter together (data not shown), which may result from the lack of microbial competition from the environment. In addition, under SEM, more yeast cells were observed localized on the inner surface of the ACTKGs than the NCTKGs (Figure 4). They seemed to not be *Y. lipolytica* since they need oxygen for growth [39]. More work needs to be done in order to give insights into this difference. However, no obvious differences were observed between the NCTKGs and ACTKGs with respect to the fine structure of grains, as well as diversity, distribution, and shift of yeast species, which demonstrated that the TKGs are relatively stable and able to compete for growth with alien microorganisms when cultivated continuously in similar milk and under similar conditions. Accordingly, culture conditions, to some extent, shape the microbial community and interaction in KGs.

In conclusion, TKGs, comprising numerous small hollow globular grain units with polyhedron-like net structures, represent one of the unusual microbial symbiotic systems in nature, which possibly resulted from a long-term symbiotic interaction among the microbial community colonizing the grains. The diversity and distribution of yeast species mainly depend on the culture milk and conditions. The *S*. *cerevisiae* and *Lactobacillus* bacteria appear to be the stable dominant microbes in the studied TKGs. These findings pave the way for further study of the specific symbiotic associations in TKGs and furthermore give insight into how the grain structure is formed.

## Supporting Information

Figure S1 FISH analysis of bacteria in TKGs. Epifluorescence micrographs of bacteria and yeasts stained with DAPI (A) and hybridization with the probe EUB338 (B) (http//131.130.66.201/probebase/) specific for most Eubacteria. (C) Merged photographs of A and B. Yeast cells are indicated with arrows. Scale bar = 10.0 µm.
(TIF)

Figure S2 FISH with pure culture strains of *Saccharomyces cerevisiae*, *Kluyveromyces marxianus*, and *Yarrowia lipolytica*. (A, C, E) Epifluorescence micrographs of yeasts stained with DAPI; (B) *S. cerevisiae* hybridized with the probe Sacch; (D) *K. marxianus* hybridized with the probes Kluyv 1 and 2; and (F) *Y. lipolytica* hybridized with the probe Ylip. Others represent merged photographs. Scale bar = 10.0 µm.
(TIF)

Table S1 Blast analysis results of cloned 26S rRNA gene sequences.
(XLSX)
