# Supplementary figures and images for: Fine Structure of Tibetan Kefir Grains and Their Yeast Distribution, Diversity, and Shift
Source: PLoS One. 2014 Jun 30;9(6):e101387. doi: 10.1371/journal.pone.0101387 (PMC4076316; doi:10.1371/journal.pone.0101387)

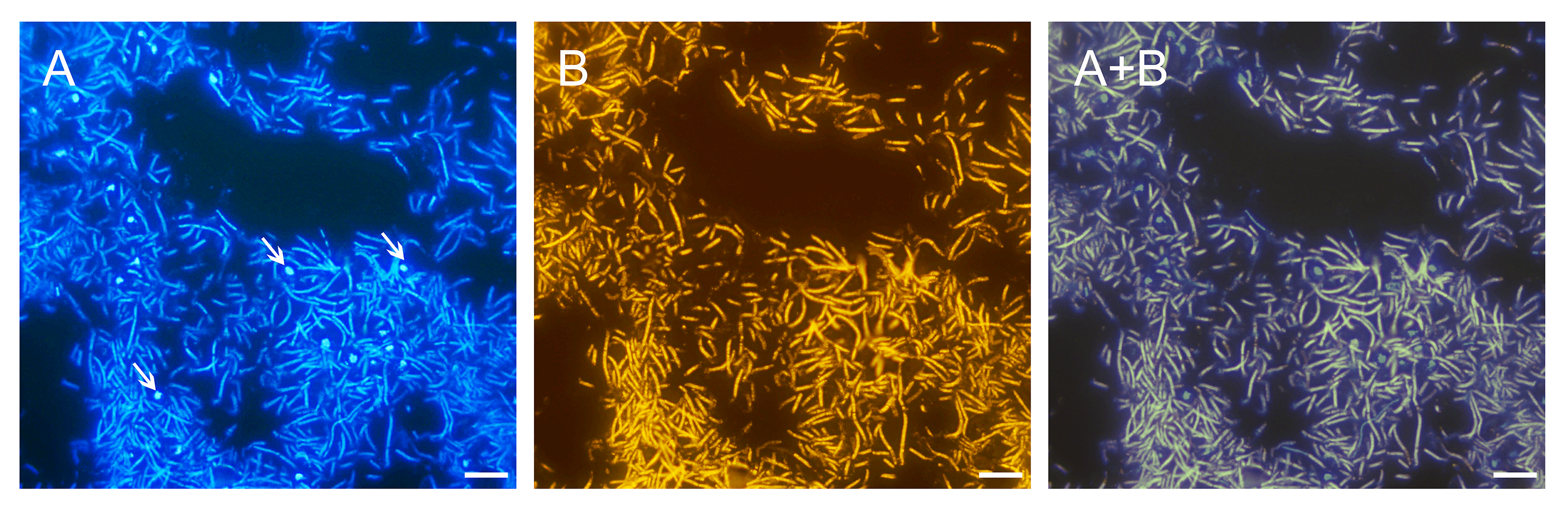

Supplement: Figure S1 — FISH analysis of bacteria in TKGs. Epifluorescence micrographs of bacteria and yeasts stained with DAPI (A) and hybridization with the probe EUB338 (B) (http//131.130.66.201/probebase/) specific for most Eubacteria. (C) Merged photographs of A and B. Yeast cells are indicated with arrows. Scale bar = 10.0 µm. (TIF) [file pone.0101387.s001.tif]

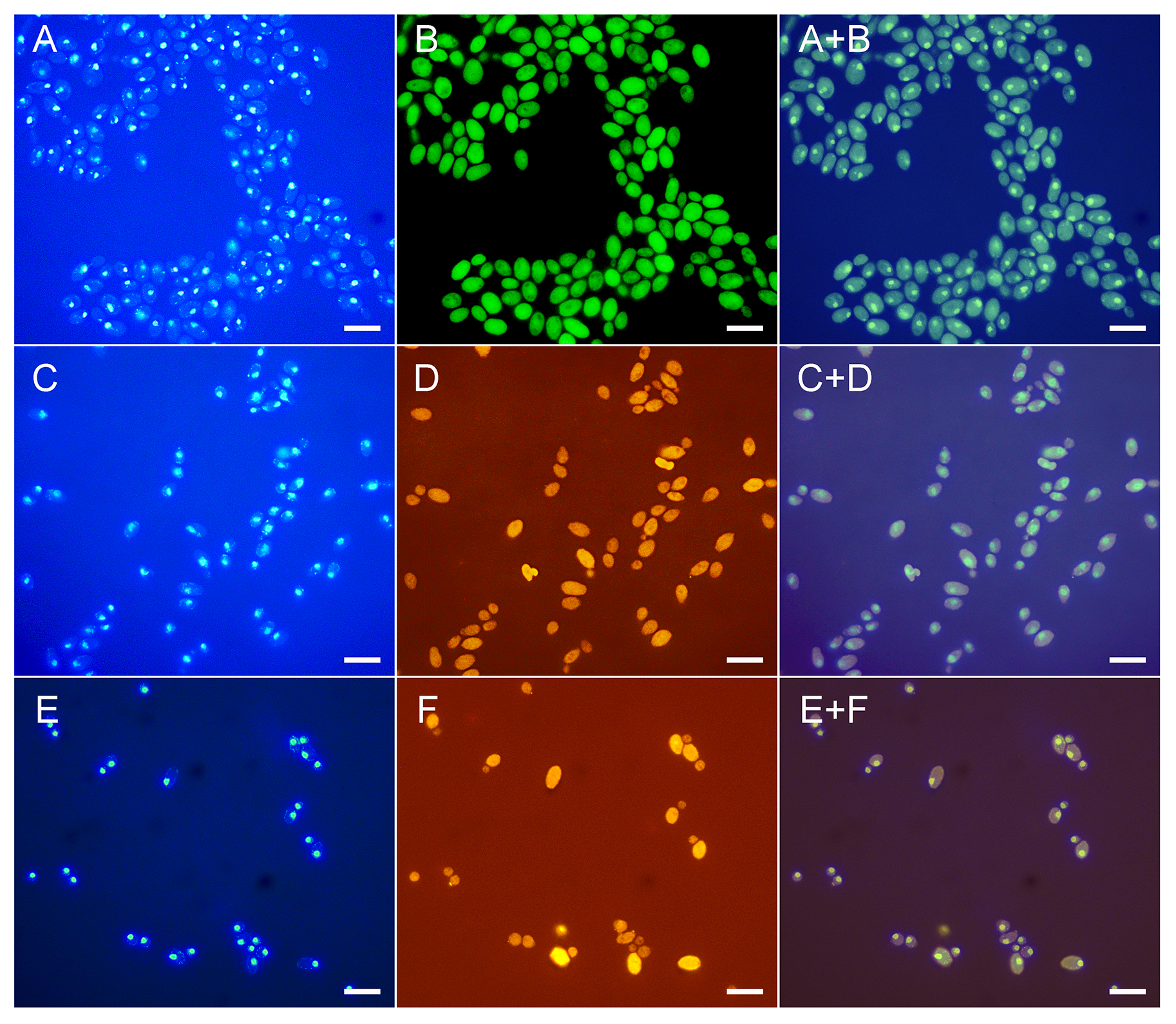

Supplement: Figure S2 — FISH with pure culture strains of Saccharomyces cerevisiae, Kluyveromyces marxianus, and Yarrowia lipolytica. (A, C, E) Epifluorescence micrographs of yeasts stained with DAPI; (B) S. cerevisiae hybridized with the probe Sacch; (D) K. marxianus hybridized with the probes Kluyv 1 and 2; and (F) Y. lipolytica hybridized with the probe Ylip. Others represent merged photographs. Scale bar = 10.0 µm. (TIF) [file pone.0101387.s002.tif]
